# Translational Medicine - doing it backwards

**DOI:** 10.1186/1479-5876-8-12

**Published:** 2010-02-08

**Authors:** Robert B Nussenblatt, Francesco M Marincola, Alan N Schechter

**Affiliations:** 1 Laboratory of Immunology, National Eye Institute, National Institutes of Health, Bethesda, MD 20892, USA; 2 Infectious Disease and Immunogenetics Section (IDIS), Clinical Center and Trans-NIH, Center for Human Immunology (CHI), National Institutes of Health, Bethesda, MD, 20892, USA; 3 Molecular Medicine Branch, National Institute of Diabetes, Digestive and Kidney Diseases, National Institutes of Health, Bethesda, MD, 20892, USA

## Abstract

In recent years the concept of "translational medicine" has been advanced in an attempt to catalyze the medical applications of basic biomedical research. However, there has been little discussion about the readiness of scientists themselves to respond to what we believe is a required new approach to scientific discovery if this new concept is to bear fruit. The present paradigm of hypothesis-driven research poorly suits the needs of biomedical research unless efforts are spent in identifying clinically relevant hypotheses. The dominant funding system favors hypotheses born from model systems and not humans, bypassing the Baconian principle of relevant observations and experimentation before hypotheses. Here, we argue that that this attitude has born two unfortunate results: lack of sufficient rigor in selecting hypotheses relevant to human disease and limitations of most clinical studies to certain outcome parameters rather than expanding knowledge of human pathophysiology; an illogical approach to translational medicine. If we wish to remain true to our responsibility and duty of performing research relevant to human disease, we must begin to think about fundamental new approaches.

NIH is the nation's medical research agency - making important medical discoveries that improve health and save lives.

*NIH is the steward of medical and behavioral research for the Nation. Its mission is science in pursuit of fundamental knowledge about the nature and behavior of living systems and the application of that knowledge to extend healthy life and reduce the burdens of illness and disability *[1].

## Editorial

A recent candidate for a post-doctoral fellowship position came to the laboratory for an interview and spoke of the wish to leave *in vitro *work and enter into meaningful *in vivo *work. He spoke of an *in vitro *observation with mouse cells and said that it could be readily applied to treating human disease. Indeed his present mentor had told him that was the rationale for doing the studies. When asked if he knew whether the mechanisms he outlined in the mouse existed in humans, he said that he was unaware of such information and upon reflection wasn't sure in any event how his approach could be used with patients. This is a scenario that is repeated again and again in the halls of great institutions dedicated to medical research. Any self respecting investigator (and those they mentor) knows that one of the most important new key words today is "translational". However, in reality this clarion call for medical research, often termed "Bench to Bedside" is far more often ignored than followed. Indeed the paucity of real translational work can make one argue that we are not meeting our collective responsibility as stewards of advancing the health of the public. We see this failure in all areas of biomedical research, but as a community we do not wish to acknowledge it, perhaps in part because the system, as it is, supports superb science. Looking this from another perspective, Young et al [2] suggest that the peer-review of journal articles is one subtle way this concept is perpetuated. Their article suggests that the incentive structure built around impact and citations favors reiteration of popular work, i.e., more and more detailed mouse experiments, and that it can be difficult and dangerous for a career to move into a new arena, especially when human study is expensive of time and money

However, pharmaceutical companies do bemoan the drying up of the therapeutic and diagnostic pipeline and the often irrelevance of *in vitro *and animal models to human disease. This has led to the marked diminution in the last several decades of the introduction of fundamental new agents into clinical medicine, despite the immense expenditures for biomedical research Indeed, it can be very readily argued that we understand the normal and abnormal states of mice more than we do human [3], and often what is known in both can be very different [4]. As Steinman wrote recently, "Animal models actually sometimes give results that are the opposite of what is ultimately seen in human disease." [5] We see such examples in recent clinical studies. A double-masked, randomized, placebo-controlled, test-of-concept trial studying the efficacy of an anti-HIV-1 vaccine aimed at eliciting cell-mediated immunity (Step Study) failed at the interim analysis after 741 vaccine and 762 placebo recipients had been treated; the vaccine did not alter the incidence of infection and infection rates tended to be higher in some treatment cohorts [6]. Unfortunately, no quality collections of human samples were included in the study to learn from this failure. This is just one of a myriad number of examples that populate the medical literature. In the case of HIV, these types of challenges have led many to call for a global vaccine enterprise, where clinical trials are better integrated with the basic science [7].

Most (with few recent potential exceptions) randomized cancer vaccine trials demonstrated poor efficacy and in some cases worse outcome in vaccinated patients [8,9]. The conclusion was that "vaccines do not work" [8], in spite of their ability to elicit cellular and immune responses which is their biological end-point. A better statement would be that vaccines do work since they reach their biological goal but we have no knowledge of the requirements in human pathophysiology that allow antigen-specific T or B cells to exert their effector function [10-12]. Such information is missing in humans simply because trials fail to study the immune response where it is most relevant: the tumor site. Thus, basic questions could not be answered: did vaccine-induced T cells reach the tumor site? Was the tumor expressing the antigen targeted by the vaccine? Why some tumors respond and some did not? We will never know unless tumor biopsies will be obtained at the right time in the course of treatment [10]. Most scientists and clinicians involved in anti-cancer immunotherapy do agree in theory that this should be done but like Sisyphus they perpetuate the enigma by following the easier path of testing the peripheral circulation or in animal models, looking over and over for the lost keys where the light is rather than where the keys were lost.

What is needed is a different template to return to the focus of our attention, the normal human state and the diseased. As Davis [3] recently noted while animal models are successful tools for understanding basic immunology they have not been successful as models of human disease. He very rightly advocates a new approach towards strategically directed efforts in human immunology. This can only mean to abandon the misnamed "Bench to Bedside" approach for a truly iterative approach with constant interplay of clinical, laboratory and even epidemiological studies. What is needed is an approach that begins at the Bedside and then goes to the "Clinical Bench" (associated studies done with patients), and finally to the animal or cellular model. It is incongruous to rely upon the use of cell or animal models if we don't know what the human pathways are. It is remarkable that a community that prides itself on facts, data, and rational thinking cannot come to address and recognize this very uncomfortable truth. At a time that genomic and other molecular approaches allow us to ask very sophisticated questions about normal and pathological processes in human beings our increasing reliance upon systems regarded as "models" for people makes no sense. A good example of observations that seem to have gone in the correct direction is that of the autoimmune lymphoproliferative syndrome (ALPS) [13]. Here the disorder, including its clinical and immunologic characteristics as well as its genetic defect, was defined in humans. This paved the way for later laboratory studies, including animal models that had relevance.

Research on model systems can bring fundamental new biological insights and animals are necessary for much work in drug development. Further, when systems in humans and animals are proven to be very similar, animal research can be very valuable in the first steps of testing new hypotheses. But such work is only part of the new conceptualization of biomedical research so urgently needed.

It has been a few years since we suggested, in the opening editorial of the *Journal of Translational Medicine*, that translational medicine is a two-way road with the bedside-to-bench direction playing a Cinderella-like role [14]. We proposed that attention to clinical realities should play a primary role in framing scientific questions according to human reality. We suggested, later on [15], that a significant impediment to the progress of biomedical research is the lack of appreciation by the current scientific establishment for descriptive, evidence-searching studies (sometimes called "omics") upon which to begin a rethinking of much biomedical research. Rather, our system is locked into testing poorly conceived hypotheses thus bypassing one of the basic elements of the scientific revolution, the Baconian principle of relevant observation and experimentation, i.e. in humans. We argued that the scientific community, while proficiently settling on the third, has progressively forgotten the first two. This has born two unfortunate results:

1) Lack of sufficient rigor in selecting hypotheses relevant to human disease to be tested in the laboratory or even later in in clinical studies.

2) Lack of sufficient rigor in conceptualizing clinical studies aimed not only at validation of therapies but also of learning from all results so as to better design subsequent trials.

Thus, we propose that hypothesis tested research should follow "facts-driven research" and only when the collection of facts relevant to human disease has been extensive, should hypotheses be constructed to expand beyond what can be directly observed.

Many naysayers will quickly come to the defense of the present system, pointing to some important advances of the last two decades. The issue is rather how efficient is our approach in meeting the NIH's goal of making important medical discoveries that improve health and save lives with its current resource base. Indeed, it can be argued that a large fraction of important observations in medicine stemmed from the clinic and laboratory work elucidating abnormal pathways. One recent example is that of trinucleotide repeats and the association with neurologic disease [16]. This seminal observation led to much fundamental research. Another was the elucidation of prions in human disease [17]. This corpus has led to enormous activity in this domain, including subsequent model studies even in yeast.

This is perhaps the crux of the need and what will be threatening to some. Clinically active physicians and non-physicians who are trained to understand human disease processes need to take a far more pro-active role in determining the paths of discovery. Today's training of physician-scientists still remains weak, in spite of efforts by the NIH and others in conceptualizing these needs. In part this is because they are being trained for niches that barely exist in many medical centers. Training based upon applications, as in engineering, would represent a significant paradigm shift for the biomedical community as a whole. However, in many ways it would be a return to the concept outlined by Stokes, where the best science in each discipline is done in the (Pasteur's) quadrant of scientific approaches most applicable, as largely the case in years past [18]. Indeed the new Director of the NIH has enunciated the need to have a stronger focus on clinical research as an important way to justify the NIH budget [19]. If we wish to remain true to our self-enunciated goals, we must begin to think about new approaches to effecting translational research.
